# Neuroendocrine adenoma of middle ear: a case report and endoscopic approach^☆^

**DOI:** 10.1016/j.bjorl.2024.101432

**Published:** 2024-04-08

**Authors:** Nicolau Moreira Abrahão, Guilherma Correa Guimarães, Jonas Belchior Tamanini, Sofia Fontes de Oliva Costa, Pedro Juliano de Mesquita Ferreira, Vagner Antônio Rodrigues da Silva, Arthur Menino Castilho

**Affiliations:** Universidade de Campinas, Faculdade de Ciências Médicas, Departamento de Otorrinolaringologia, Campinas, SP, Brazil

## Introduction

Neuroendocrine Adenoma of the Middle Ear (NAME) is a rare and a benign cause of retrotympanic mass^1^, ^2^ accounting for fewer than 2% of middle ear tumors.^1^ It is originated from a neuroendocrine differentiation of middle ear epithelium and for its diagnosis is mandatory to perform a histology and an immunohistochemistry studies as the clinical presentation, otoscopy and radiological findings are non-specific. Complete surgical removal is the treatment of choice.^2^

## Case report

A 31-year-old female complaining of progressive hypoacusis and pulsatile tinnitus on the right ear, started 2-years ago, with no otorrhea or dizziness. The otoscopy examination on the left ear was normal. An intact tympanic membrane bulged by a reddish retrotympanic mass was visualized on the right ear.

**Figure 1:**
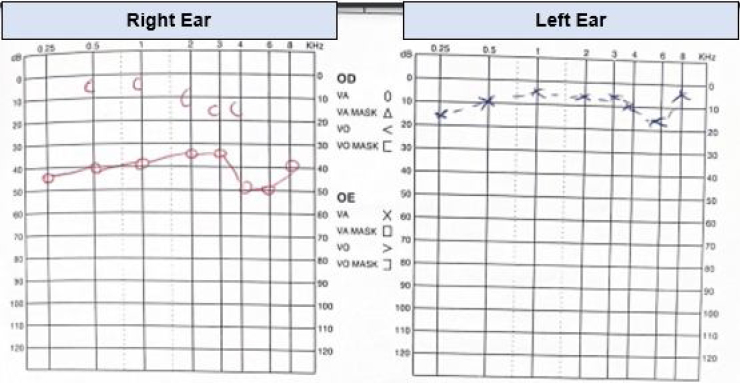
Audiometry demonstrating moderate conductive hearing loss in right ear.

The audiometry demonstrated right moderate conductive hearing loss, with pure tone average (PTA, calculated at 0.5, 1, 2, 4 kHz) of 60 dB HL on the right ear. Speech audiometry confirmed right ear hearing loss (Fig. 1).

**Figure 2:**
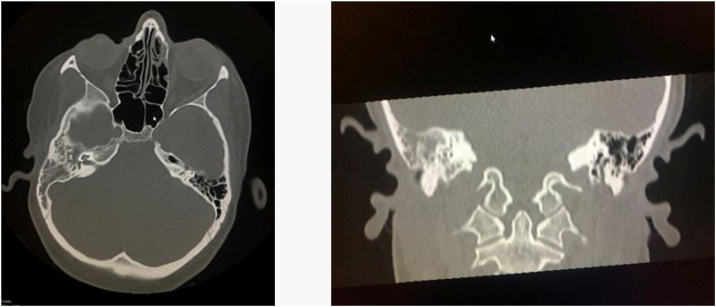
CT-scan of temporal bone demonstrating a hypointensity tissue filling all the middle ear and epytimpanic space.

A Computed Tomography of temporal bone (CT) demonstrated a hypointensity tissue filling all the middle ear and epytimpanic space and obstructing the eustachian tube orifice on the right ear. The ossicular chain is disrupted and eroded by the mass effect (Fig. 2).

**Figure 3:**
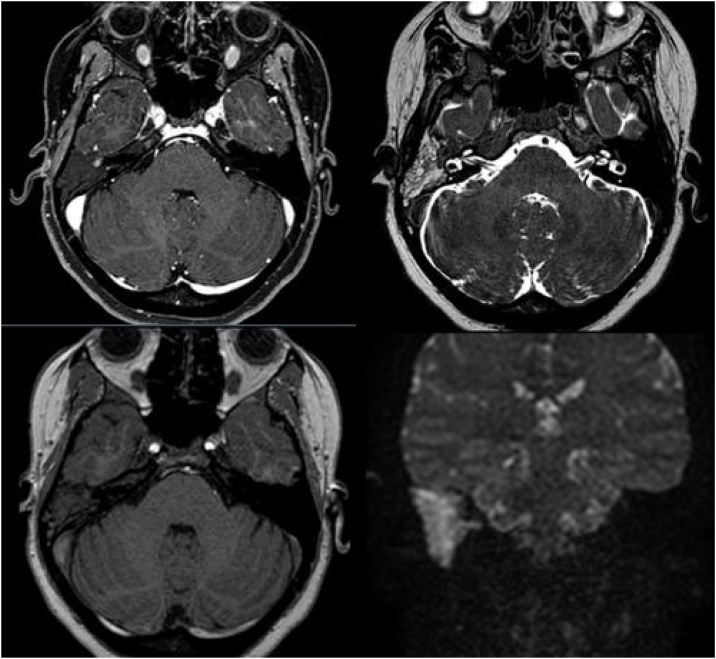
MRI demonstrating an iso-intense mass filling the mesotympanum, in contact with the internal carotid artery, with no evidence of bone erosion.

A Magnetic Resonance Imaging (MRI) showed an iso-intense mass filling the mesotympanum, expanding in the eustachian tube, in contact with the internal carotid artery channel, but with no evidence of bone erosion, not showing enhancement after gadolinium administration. A liquid was filling the epytimpanic space, the aditus ad antrum, and the mastoid cells as seen in the T2 phase (Fig. 3).

**Figure 4:**
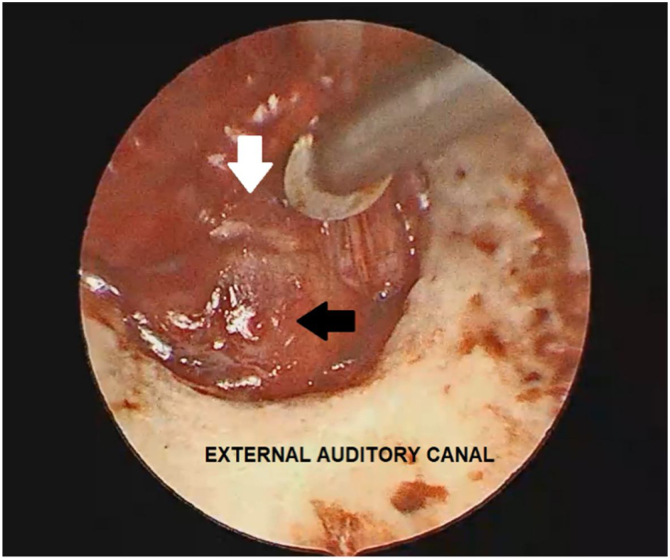
The endoscopic view: after the tympanomeatal flap was elevated, the grey dissector is softly separating the tumor (black narrow) to the tympanic membrane annulus (white narrow).

In the current case, the authors have decided to remove the tumor surgically, and the Endoscopic Approach (EA) was chosen as it was limited to the middle ear and did not extend to the mastoid half of the lateral semicircular canal. A tympanomeatal flap was raised from 6 o’clock to 12 o’clock up to access the middle ear and then a reddish mass, occupying the hypotympanum and the mesotympanum with an important adhesion to the promontory and the ossicular chain was visualized (Fig. 4).

**Figure 5:**
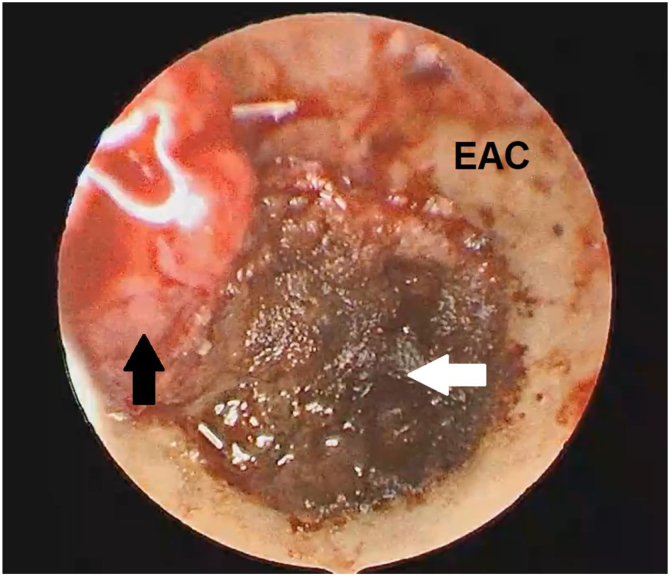
The endoscopic view: the black narrow shows an intact tympanic membrane instead the white narrow shows the tumor after the bipolar cautery is used to downsize the mass. EAC, External Auditory Canal.

The gentle movement was performed to separate the tympanic membrane from NAME. The bipolar cautery and adrenaline cotton balls were used to downsize the mass (to reduce the bleeding) then the mass was easier removed in pieces, with microforceps and microhooks dissector (small and large) with no surgical complications (Fig. 5).

**Figure 6:**
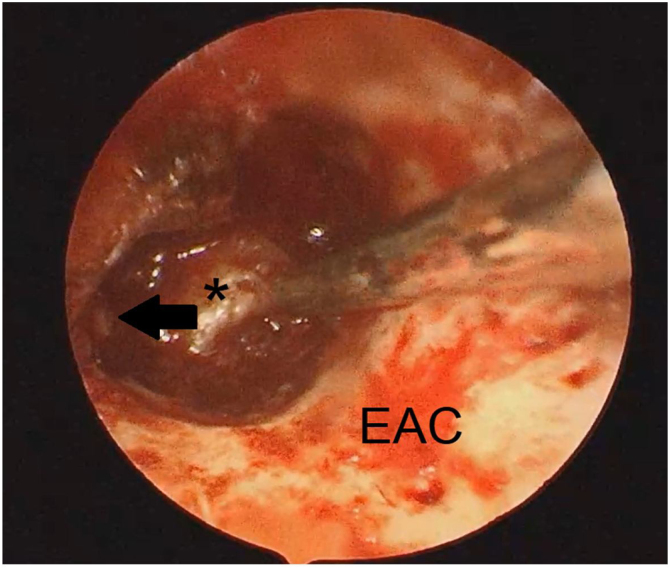
The endoscopic view: the middle ear space clear of disease after the mass was excised. The black narrow shows the stape superstructure. *, Promontory EAC, External Auditory Canal.

After been removed from the mesotympanum the promontory was clearly visualized (Fig. 6). The ossicular chain was removed because the tumor was adhered in it and is demonstrated that, in this situation, if the ossicular chain is not removed the risk of recurrence is higher.^2^

**Figure 7:**
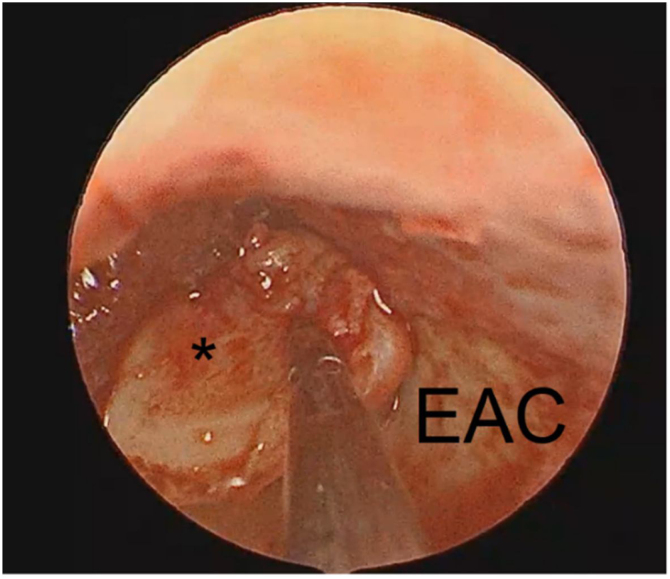
The endoscopic view: the middle ear reconstruction with tragus cartilage (*). EAC, External Auditory Canal.

After the tumor was removed, the material was sent to pathological analysis that demonstrated, through the histology and immunohistochemistry, a NAME pattern. The middle ear space was filled with surgicel and the tragus cartilage was used to rebuild the tympanic membrane (Fig. 7).

## Discussion

The first description of Neuroendocrine Adenoma of the Middle Ear (NAME) as pathology was made in 1976 by Hyams and Michaels.^3^ NAME emerges from a neuroendocrine differentiation of middle ear epithelium, and its histological pattern shows a cuboid, and cylindrical cells that have well defined boundaries, eosinophilic cytoplasm, and hyperchromatic round nuclei, without mitotic figures.^4^

The clinical presentation can widely vary between patients. The most common symptoms are hearing loss, aural fullness, vertigo, tinnitus, bleeding, infections and pain.^4^ The macroscopic appearance is poorly vascularized, has a grey or reddish-brown color and a fibrotic consistency not being specific instead.^4^ The CT-scan examination of the temporal bone usually shows a homogenous, hypodense lesion well limited to the middle ear.^5^ The ossicular chain erosion, and facial nerve involvement should be excluded. MRI reveals an iso-intense mass during T1 with homogenous enhancement following the injection of gadolinium.^5^

As described in the literature, the clinical presentation, otoscopy and radiological findings are non-specific^5^ being mandatory a histology and immunohistochemistry studies. Besides the literature suggests that NAME are benign, well-localized tumors that do not metastasize. It should be managed by surgery.^5^

## Conclusion

Neuroendocrine Adenoma of Middle Ear (NAME) is a rare and a benign cause of retrotympanic mass.^1^, ^2^ The treatment of choice is surgery^2^ that allows the removal of the tumor and the harvest material to a histology and an immunohistochemistry studies, that are mandatory to exclude other pathologies of the middle ear. The authors have performed an endoscopic approach to remove the tumor and rebuild the tympanic membrane with tragus cartilage in the current case.

## Funding

There is no financial or material supports.

## Conflicts of interest

The authors declare no conflicts of interest.
